# Breast-Related Herniation: A Call for Multidisciplinary Awareness and a Proposal for a Classification

**DOI:** 10.7759/cureus.75825

**Published:** 2024-12-16

**Authors:** Rami Yaghan, Nehad M Ayoub, Lamees R Yaghan, Eyad A Baqain, Shadi Hamouri, Abdulla I Mohamed

**Affiliations:** 1 Department of Surgery, Arabian Gulf University, Manama, BHR; 2 Department of Surgery, Jordan University of Science and Technology, Irbid, JOR; 3 Department of Clinical Pharmacy, Jordan University of Science and Technology, Irbid, JOR; 4 Medical Skills and Simulation Center, Arabian Gulf University, Manama, BHR

**Keywords:** breast cancer, breast herniation, breast infection, classification, hernia, tuberous breast, yaghan hernia

## Abstract

Breast-related herniation (BRH) is a vague term for many clinicians. The absence of a universal nomenclature and the different nature of the herniation process involved, being true or false, contribute to this vagueness. BRH includes a spectrum of disorders ranging from a few congenital breast disorders to commoner herniation processes related to acquired breast diseases. We aim to raise multidisciplinary awareness about BRH by reviewing the related literature and reviewing our experience with BRH. We will also propose a classification for BRH. PubMed and Scopus databases were searched for any herniation disorders that are related to the breast in any clinical or pathological aspect. The literature review revealed 12 various groups of BRHs that we could classify into two anatomical categories: pectoral (mammary) BRH, in which the herniations occur at the pectoral site of the chest wall, and extra-pectoral BRH, in which the herniations occur at an extra mammary site but are related to breast pathologies. Pectoral BRH was further divided into clinicopathological subcategories, including congenital BRH, pulmonary BRH complicating breast cancer and breast infections, factitious BRH, and iatrogenic BRH. Extra-pectoral BRH was further divided into clinicopathological subcategories, including abdominal wall hernias following autologous breast reconstruction (ABR) and herniated siliconomas. Our study group included 19 patients with BRHs, among which congenital BRHs and pulmonary BRHs were the most common. Congenital BRHs are rare and are of main interest to plastic surgeons. However, abdominal wall hernias following ABR, pulmonary BRH, and factitious BRHs are relatively common complications of breast cancer treatment. This calls for a multidisciplinary approach to ameliorate the morbidity of BRHs. Further studies are needed to refine our proposed classification.

## Introduction

Breast-related herniation (BRH) is a vague entity for most clinicians. The absence of a universal nomenclature contributes to this vagueness. BRHs vary from true anatomical hernias to false hernia-like lesions. Pathologically, BRHs include a wide spectrum of disorders ranging from rare congenital breast disorders such as the so-called herniation of the nipple-areola complex (HNAC) or tuberous breast (TB) [1] to the relatively common acquired herniation processes related to breast cancer and breast infections [2-4]. Although the treatment of BRHs is the primary responsibility of surgeons, awareness among other multidisciplinary breast team members might ameliorate the morbidity of BRHs [5], particularly those related to breast cancer.

We aim to raise multidisciplinary awareness about BRH by reviewing the related literature and reviewing our experience with BRH. We will also propose a clinicopathological classification for BRH. This classification will facilitate communication between physicians and will be the first step toward a universal nomenclature for BRH.

The study was conducted at the Faculty of Medicine at Jordan University of Science and Technology and its affiliated King Abdulla University Hospital (KAUH)-Jordan and the College of Medicine and Health Sciences at Arabian Gulf University-Bahrain between January 2007 and December 2023. The Institutional Review Board at KHUH approved the study (number 518-2017). Informed consent was waived in view of the retrospective and anonymous nature of data collection.

## Technical report

Definitions

In this study, we introduced the term BRH to indicate all true or false herniation processes that are related to the breast in any clinical or pathological aspect. True herniation indicates the presence of the classical components of a hernia, i.e., there is a recognized anatomical defect or weakness through which a reducible herniation process is taking place. False herniation refers to hernia-like lesions that lack the classical components of a hernia.

Literature review

PubMed and Scopus were searched using different combinations of the following keywords: "breast", "hernia", and "herniation." The final search was conducted on November 10, 2024. All types of publications were included. A total of 974 publications in PubMed and 1,706 publications in Scopus were initially retrieved. The titles and abstracts of these publications were screened by two authors independently (R.Y. and A.M.).

Inclusion Criteria

Publications with specific data relevant to our definition of BRH were included. Recent systematic reviews and meta-analyses were given priority whenever available.

Exclusion Criteria

Publications lacking data relevant to our definition of BRH were excluded.

After the application of the above inclusion and exclusion criteria, the two authors (R.Y. and A.M.) agreed on 42 publications. The full texts of these 42 publications constituted the final basis for writing the review and proposing the classification of BRH.

Results of the iterature review

We identified 12 groups of disorders that fulfill our definition of BRH (Table 1).

**Table 1:** BRH in a descending trend of the frequency of occurrence AAWHB: anterior abdominal wall hernia/bulge; ABR: autologous breast reconstruction; BRH: breast-related herniation; IHRCW: implant herniation related to capsular weakness; LDMF: latissimus dorsi myocutaneous flap; S: siliconoma; TB: tuberous breast

Group number	Disorder
1	AAWHB following ABR
2	TB
3	IHRCW
4	Pulmonary BRH complicating breast cancer
5	Localized S
6	Herniated S
7	Pulmonary BRH complicating breast infection
8	Lumbar hernia following the harvest of LDMF
9	Intrapleural implant herniation
10	Epigastric hernia following ABR
11	Iatrogenic BRH
12	Yaghan breast herniation

Anterior abdominal wall hernia/bulge (AAWHB), following autologous breast reconstruction (ABR), was the commonest BRH. In one of the biggest prospective trials for abdominal flap-based ABR from 11 centers, the two-year AAWHB rate was 3.1% [6]. TB was the second most encountered BRH, although the exact prevalence in the general population was difficult to assess [7]. Implant herniation related to capsular weakness (IHRCW) was the third most encountered BRH, although capsular weakness may have been underreported in the literature [8,9]. Pulmonary BRH complicating breast cancer [10] and localized and herniated siliconomas (Ss) [11] were rare complications of breast cancer treatment. The remaining BRHs (numbers 7-12 in Table 1 ) were encountered as limited case series or isolated case reports and included pulmonary BRH complicating breast infection [4], lumbar hernias following the harvest of latissimus dorsi myocutaneous flaps (LDMFs) [12], intrapleural breast implant herniation [13-18], epigastric hernias following ABR [19], iatrogenic BRH [20], and Yaghan breast hernia [21]. In the discussion section, we will highlight the multidisciplinary relevance of the commonest BRHs [6-11], as encountered in our literature review. The remaining quite rare BRHs (numbers 7-12 in Table 1) are briefly mentioned for the sake of comprehensiveness of our proposed classification.

Authors’ experience

Table 2 summarizes the pertinent demographic and clinicopathological data of 19 patients with BRHs who were treated at KAUH between January 2007 and December 2023. One of these cases has been reported previously [21]. Among our study group, TB and pulmonary BRH complicating breast cancer were the most encountered BRHs. The specific features of our patients are highlighted in the related clinicopathological BRH subcategories in the discussion.

**Table 2:** Patients presenting with BRH at KHUH from 2007 to 2023 (N = 19) BRH: breast-related herniation; KAUH: King Abdulla University Hospital; S: siliconoma; TB: tuberous breast; †median (interquartile range), ‡for female patients only

BRH	n	Gender	Age at diagnosis, years
TB	13	Female 12, Male 1	19.5 (17.5-21.75)^ †‡^
Pulmonary BRH complicating breast cancer	4	Female 4	54.5 (42.3-59.25)^ † ^
Yaghan breast hernia	1	Female 1	28
Localized S	1	Female 1	50

Classification of BRH

Our literature review revealed 12 groups of disorders that fulfill our definition of BRH (Table 1). We could classify these 12 groups of BRHs into two anatomical categories (Table 3): pectoral (mammary) BRH and extra-pectoral BRH. The pectoral BRH category includes herniations that occur at the pectoral (mammary) site of the chest wall and might involve the breast parenchyma, the breast fascial covering, or the underlying lung and pleural tissue through a defect in the sub-mammary chest wall. The extra-pectoral BRH category includes herniations occurring at an extra-pectoral site away from the breast but are related to breast diseases. The two anatomical categories of BRHs were further divided into clinicopathological subcategories (Table 3).

**Table 3:** Proposed clinicopathological classification of BRH AAWHB: abdominal wall hernia/bulge; ABR: autologous breast reconstruction; BRH: breast-related herniation; IHRCW: implant herniation related to capsular weakness; LDMF: latissimus dorsi myocutaneous flap; S: siliconoma, TB: tuberous breast

Anatomical category	Clinicopathological category		Etiology	Nature
Pectoral (mammary) BRH	A. Tuberous breast		Congenital	False
	B. Yaghan breast Herniation		Congenital	True
	C. Pulmonary BRH	a. Pulmonary BRH complicating breast cancer	Acquired	True
		b. Pulmonary BRH complicating breast infection	Acquired	True
	D. Factitious BRH	a. IHRCW	Acquired	True
		b. Localized S	Acquired	False
		c. Intrapleural implant herniation	Acquired	True
	E. Iatrogenic BRH		Acquired	True
Extra-pectoral BRH	A. Abdominal wall herniation following ABR	a. AAWBH following ABR	Acquired	True
		b. Lumbar hernia following the harvest of LDMF	Acquired	True
		c. Epigastric hernia following ABR	Acquired	True
	B. Herniated S		Acquired	False

## Discussion

This is the first attempt to review BRHs under one theme in a multidisciplinary conceptual approach. Although the treatment of BRHs is the primary responsibility of surgeons, awareness among other breast team members might ameliorate the morbidity of some BRHs, particularly those related to the treatment of breast cancer, such as AAWHB, pulmonary BRH, and factitious BRHs. A multidisciplinary approach will provide the non-surgical treating members with an overview of the challenges faced by the surgeons while treating BRHs. By the same token, surgeons will have a better insight into how the nonsurgical treatment modalities of breast cancer might affect the selection of the optimum surgical option. For example, the incidence of AAWHB was found to be higher in patients receiving chemotherapy for advanced stages of breast cancer [5] and in patients receiving letrozole in the perioperative period [22]. Accordingly, the surgeon might modify the type and timing of breast reconstruction, aiming for the least possible morbidity and the best patient satisfaction and quality of life. In general, the impact of the morbidity of BRH seems to be underestimated by the clinicians. We have conducted this study to raise multidisciplinary awareness about BRHs and highlight the clinical implications, which are of importance to all clinicians involved in the treatment of breast disorders. There is a clear need for a universal nomenclature and classification for BRHs to facilitate such awareness. Our proposed classification for BRHs is the first step in this direction and is based on anatomical and clinicopathological parameters (Table 3). We call for further insights in this context.

The following review addresses the multidisciplinary aspects of BRH. The surgical technical details are outside the realm of this study.

Tuberous breast

TB is the most enigmatic category of BRH. TB, first described by Rees and Aston in 1976, is related to the failure of proper development of the base of the breast while the central parenchyma continues to grow and prolapses anteriorly, resulting in the so-called HNAC [1]. Obviously, this is a false herniation and is a historical misnomer. The nipple-areola complex (NAC) will get hypertrophied with areolar widening [1]. Other names were given to TB, including tubular breast, narrow-based breast, snoopy deformity, and lower-pole hypoplastic breast, adding to its confusing terminology [21]. TB usually manifests during puberty with significant psychological and cosmetic morbidity [1]. There is a clear need for proper psychological counseling for these patients.

The exact etiology of TB is not known. Most reports relate TB to the presence of dense congenital constricting fibrous rings at the base of the breast, leading to deficient horizontal and vertical development of the base of the breast and breast hypoplasia [1]. Six cases of TB were reported in consanguinity, suggesting a possible genetic role in some patients with TB [23].

The prevalence of TB is difficult to assess. Klinger et al. showed that 48.5% of their patients presenting for breast augmentation, 47.3% of their patients presenting for breast reductive surgery, and 27.6% of the general population demonstrated at least one deformity related to TB [7]. We agree that the prevalence of TB is underestimated. Mild degrees of TB might pass unnoticed by both doctors and patients [24]. Social constraints in conservative societies might be another factor. Adolescent girls in these communities might be reluctant to report their concerns about their breast shape. This is evident in our series, in which the median age at presentation was delayed until 19.5 years (Table 2). Our series included one male patient with full-blown TB and gynecomastia (Table 2). This is rare. In fact, most male patients with TB present with minor forms of NAC over-projection with associated gynecomastia and a normal base of the breast [25,26]. Surgery in this subgroup of gynecomastia patients mandates extra procedures for optimum results.

TB is the only BRH with previous technical surgical classifications. The availability of at least eight of these classifications reflects the complexity of the surgical treatment of TB [1,27-30]. These detailed classifications are beyond the multidisciplinary target of this report but remain invaluable for plastic surgeons to plan the surgical treatment of TB.

Yaghan breast herniation

Yaghan et al. reported the case of a 28-year-old female patient who presented to our center with a reducible mass in the lower half of her left breast [21]. This was due to herniation of the part of the left breast through a congenital defect in the superficial fascia of the anterior thoracic wall, which normally invests the breast. Primary closure of the defect produced a satisfactory outcome [21]. This was the only reported true congenital breast hernia. All the anatomical and clinical elements of a classical surgical hernia were present.

Pulmonary BRH

In this paper, pulmonary BRH refers to the protrusion of the pleural membranes or the lung parenchyma through a defect or weakness in the pectoral thoracic wall as a complication of breast cancer or extensive lung and breast infection. The herniating lung tissue might be covered with the weakened chest wall or might be exposed with a flimsy pleural cover.

Pulmonary BRH Complicating Breast Cancer

Breast cancer is the most common cause of pulmonary BRH. The weakness in the pectoral area can be the result of the local treatment of breast cancer by mastectomy and radiotherapy leading to radio-necrosis of the ribs, the local recurrence of the disease, or the aggressive nature of the primary tumor [10]. Patients present with a bulge in the mammary region, which increases in size during coughing with occasional flail chest presentation. Treatment consists of resection of the affected part of the chest wall followed by major reconstruction [3]. A multidisciplinary approach is crucial to individualize the extent of reconstructive surgery according to the severity of symptoms, the pre and post-reconstruction performance status, and the expected survival. Our series included four patients in this subcategory (Table 2). Two patients had stage IV terminal breast cancer with limited expected survival. Major reconstructive chest wall surgery was not justified. The other two patients underwent major chest wall reconstruction. 

Pulmonary BRH Complicating Breast Infection

Empyema necessitans is a rare clinical entity in which an intrathoracic empyema decompresses through the parietal pleura and chest wall, forming a collection of pus in the extra-thoracic soft tissues [4]. If this happens in the vicinity of the breast, pulmonary BRH might occur through the weakened or damaged chest wall [4]. Mycobacteria and actinomycosis are the commonest pathogens [31]. Inversely, primary breast infections by these organisms can damage the pectoral chest wall leading to pulmonary BRH [31,32].

Factitious BRH

This clinicopathological subcategory includes BRHs related to complications of silicone breast implants.

IHRCW

Capsule formation, or its advanced form capsular contracture, is the most recognized long-term complication of breast implants occurring at a range of 8-41% [8]. The currently reported incidence of explanation of breast implants is around 12% at a median time of 1.6 years, with cosmetic dissatisfaction, capsular contracture, and pain being the most common indications [11]. A less recognized complication is the capsule weakness in which the capsule is thin and lax. Capsular weakness can lead to herniation of the implant (or part of it) in the subcutaneous tissue [8,9] or what we call IHRCW. Careful palpation gives the feeling of a reducible smooth swelling or, in fact, a hernia. Occasionally, total implant herniation (displacement) into the axilla, or the upper abdomen necessitates a redo surgery [8].

Dale et al. reported a case of IHRCW because of periprosthetic tuberculosis [33]. A rising rate of periprosthetic mycobacterial infections in developing countries has been reported [34]. *Mycobacterium fortuitum* was the most common isolated species [34].

Silicone granulomas (Ss)

An S, or a silicone granuloma, is a tender subcutaneous nodule resulting from the reaction of the body to silicone deposits from ruptured breast implants. This can occur in the vicinity of the implant (localized S) or much less likely, silicone gel migration can occur to extra pectoral sites, such as axillary lymph nodes, upper arms, inguinal areas, and lower limbs producing the so-called herniated or migratory Ss [35,36] (Table 3).

The mechanism of remote migration is not sufficiently understood. Ss migrating to hernial sites can very easily be misdiagnosed as strangulated hernias. Such a mishap might be the first indication of an existing yet unrecognized ruptured breast implant. MRI is the best tool to assess breast implants [35]. Histological examination of Ss reveals mixed cell foreign body granulomas with cystic vacuoles containing silicone [35]. Scanning a specimen of the resected tissue by electron microscopy is a helpful diagnostic test in controversial cases [36]. We recommend complete surgical removal of Ss given the still unresolved possible association of silicone implants (and logically Ss) with Sjogren syndrome, scleroderma, rheumatoid arthritis, melanomas, and lymphomas [37]. It is reassuring that the vast majority of Ss were reported in prosthetics manufactured decades ago [35]. Strict manufacturing regulations have brought the number of Ss to a minimum.

Intrapleural Implant Herniation

There are a few reports about the herniation of breast implants into the pleural cavity. These herniations were mainly reported after open or video-assisted thoracic surgeries in patients with previous implant-based breast reconstruction [13-15,18]. Obviously, the herniations were precipitated by post-surgical weakness of the chest wall. However, intrapleural implant herniation was also reported without any chest wall surgeries, probably due to the repeated pressure by the implant on the underlying chest wall muscles [16,17]. Fong et al. reported the case of a 59-year-old female patient who underwent bilateral post-mastectomy implant-based reconstruction. She reported that “her body swallowed one of the implants during a Pilates stretching exercise” [17]. She presented with ballooning of her right anterior chest wall during the Valsalva maneuver. CT scan revealed the herniation of the right prosthesis in the pleural cavity.

Apart from causing diagnostic dilemmas, the herniated devices in the pleura could be removed, and the defect in the chest wall was corrected without significant morbidities [13-18].

Iatrogenic BRH

An astonishing case of post-ductography external herniation of an intraduct papilloma has been reported [20].

Abdominal wall herniation following ABR

Abdominal wall integrity may be compromised after abdominal myocutaneous flap harvesting for ABR, leading to abdominal wall hernia/bulge.

AAWHB Following ABR

Donor-site AAWHB was the most encountered BRH. Historically, the pedicled transverse rectus abdominis myocutaneous ﬂap (pTRAM) was the most popular flap and was associated with the highest rate of AAWHB [2]. Efforts by plastic surgeons to reduce the morbidity of ABR and improve patient satisfaction led to the introduction of further flaps such as the free transverse rectus abdominis myocutaneous ﬂap (fTRAM), deep inferior epigastric perforator flap (DIEP), and superﬁcial inferior epigastric artery perforator flap (SIEA). A recent systemic review and meta-analysis, including a total of 37 studies, compared the complications and donor site morbidities of pTRAM, fTRAM, DIEP, and SIEA [2]. SIEA was associated with the lowest risk of AAWHB [2]. In keeping with this, Knox et al. found that pTRAM was associated with a 21.2% AAWHB compared to 3.1% in DIEP [38]. However, DIEP reconstruction required an average extra operative time of 234 minutes.

The addition of mesh during abdominal wall closure reduces the incidence of AAWHB [39]. There are no universal guidelines for the use of mesh, type of mesh, or plane of mesh placement. The preponderance of evidence is however in favor of using polypropylene mesh [39, 40].

Nipple and skin-sparing mastectomy (NSM) with implant-based reconstruction is an option that abolishes AAWHB. The current evidence indicates the oncological safety of NSM in properly selected patients [41]. Our series did not contain any AAWHB. This is because our center is the recognized regional center for NSM. Patients in need of ABR are referred to another center.

Patel et al. demonstrated an increase in the incidence of AAWHB in patients undergoing chemotherapy for advanced stages of breast cancer [5]. This was most pronounced in patients undergoing bilateral reconstruction and in patients with preexisting DM. Hubor et al. reported an increased rate of AAWHB (13.5%) among patients who received letrozole in the perioperative period [22]. In animal models, tamoxifen administration resulted in the appearance of a large lower abdominal bulge in male rats [42]. The impact of anti-estrogen treatment on hernia formation, if any, requires further studies.

In brief, AAWHB can interfere with the physical and cosmetic well-being of patients. A multidisciplinary approach is essential to offer the optimum surgical procedure for each patient.

Lumbar Hernia Following the Harvest of LDMF

Lumbar hernias were reported as rare complications of ABR using LDMFs [12]. Patients present with swelling in the lumbar region a few months after surgery. The hernia may contain retroperitoneal fat, kidney, or colon. Lumbar ultrasound or CT scans are effective tools in terms of diagnosis and surgical planning. Small defects might be closed primarily [12]. Larger defects necessitate reinforcement by prosthetic mesh, preferably by laparoscopic approach, for optimal exposure and shorter hospital stays.

Epigastric Hernia Following ABR

Conroy et al. reported three epigastric hernias after DIEP breast reconstruction [19]. They concluded that although the association with DIEP may have been purely coincidental, abdominal flap harvest might have caused weakness of the upper anterior abdominal wall.

Limitations of the study

The introduction of six new terms (pectoral BRH, extra pectoral BRH, pulmonary BRH, factitious BRH, IHRCW, and iatrogenic BRH) creates an initial difficulty for readers. Understandably, this was necessary to address BRHs under one theme and propose a classification. There were no previous clinicopathological terms for these lesions. 

## Conclusions

This is the first attempt to review BRHs under one theme in a multidisciplinary conceptual approach. The literature review revealed 12 distinct groups of BRHs that we classified into two anatomical categories: pectoral (mammary) BRH and extra-pectoral BRH. Pectoral BRH included the following clinicopathological subcategories: congenital BRHs, pulmonary BRHs complicating breast cancer and breast infections, factitious BRHs, and iatrogenic BRH. Extra-pectoral BRHs included the following clinicopathological subcategories: abdominal wall hernias or bulges complicating ABR and herniated Ss. Congenital BRHs are rare and are of main interest to plastic surgeons. However, abdominal wall hernias complicating ABR, pulmonary BRH, and factitious BRHs are relatively common complications of breast cancer treatment. This calls for a multidisciplinary approach to ameliorate the morbidity of BRHs. Further correspondence is essential for the refinement of our proposed terminology and classification.
